# Update of guideline for diagnosis and treatment of subacromial pain syndrome: a multidisciplinary review by the Dutch Orthopedic Association: Part 2: Operative considerations and treatment of various conditions related to subacromial pain syndrome

**DOI:** 10.2340/17453674.2026.45410

**Published:** 2026-02-16

**Authors:** Frederik O LAMBERS HEERSPINK, Egbert J D VEEN, Oscar DORRESTIJN, Cornelis P J VISSER, Maarten J C LEIJS, Dennis VAN POPPEL, Peter A STROOMBERG, Ramon P G OTTENHEIJM, Jan W KALLEWAARD, Tjerk J W DE RUITER, Henk A MARTENS, Femke M JANSSEN, Tessa GELTINK, Matthijs S RUITER, Jos J A M VAN RAAIJ

**Affiliations:** 1 Department of Orthopaedics, Viecuri Medical Center, Venlo; 2 Department of Orthopaedics and Research School Caphri, Maastricht University Medical Centre+, Maastricht; 3 Department of Orthopaedics, Medical Spectrum Twente, Enschede; 4 Department of Orthopaedics, Sint Maartens Clinic, Nijmegen; 5 Department of Orthopaedics, Alrijne and Eisenhower Clinic, Leiderdorp; 6 Department of Orthopaedics, Reinier Haga Orthopedic Center, Zoetermeer; 7 Department of Physical Therapy, PECE Zorg, Fontys, Eindhoven; 8 Department of Radiology, Isala clinic, Zwolle; 9 Department of General Practice, Maastricht University, Maastricht; 10 Department of Anesthesiology, Rijnstate Clinic, Arnhem; 11 Department of Rehabilitation, De Ruiter Rehabilitation, Hengelo; 12 Department of Rheumatology, Sint Maartens Clinic, Nijmegen, the Netherlands; 13 Knowledge Institute of the Federation of Medical Specialists, advisor, Utrecht; 14 Department of Orthopaedics, Martini Hospital Groningen, Groningen, the Netherlands

## Abstract

**Background and purpose:**

In 2013, the first clinical practice guideline for subacromial pain syndrome (SAPS) was developed in the Netherlands to support healthcare professionals. SAPS refers to non-traumatic, non-rheumatologic shoulder complaints that are particularly painful during arm elevation. It includes conditions such as supraspinatus tendinosis, calcific tendinitis, and degenerative supraspinatus tears. Over 50,000 patients annually consult orthopedic surgeons for these issues. In response to new evidence and clinical needs, an updated guideline was developed. Part 2 focuses on supraspinatus tears, biceps tendon pathology, and calcific tendinosis. Using a multidisciplinary, evidence-based approach, the guideline aims to answer key clinical questions around SAPS.

**Methods:**

Initiated by the Dutch Orthopedic Society, the guideline committee identified knowledge gaps through group sessions. Each module was based on a PICO-formatted key question and reviewed by professionals from different fields. The AGREE and GRADE methods were applied to ensure a systematic evaluation of evidence, leading to conclusions and recommendations.

**Results:**

(i) Start with exercise-based therapy (with corticosteroid injection) for isolated, symptomatic, non-traumatic supraspinatus tears. Consider cuff repair if no improvement after 3–6 months. (ii) Avoid biceps tenotomy/tenodesis on a healthy tendon unless at risk during cuff repair. (iii) Evaluate patient- and tear-specific factors; use MRI for detailed assessment. (iv) Consider barbotage for calcific tendinosis; repeat once if needed. Reserve surgery for persistent large calcifications. (v) Postoperative immobilization should not exceed 3 weeks.

**Conclusion:**

The updated guideline provides multidisciplinary recommendations for surgical management.

Subacromial pain syndrome (SAPS) is a common cause of shoulder pain. To optimize the treatment and minimize practice variation, a multidisciplinary guideline for SAPS was developed. In part 1 we focused on preventive measures, diagnostics, and non-surgical treatment of SAPS [1]. In this part, we present guidelines concerning operative treatment of various conditions related to SAPS. In 2021, the module on surgical treatment for SAPS was updated, strongly recommending against subacromial decompression surgery. This recommendation is based on scientific evidence showing that surgery offers no additional benefit over non-surgical treatment [2-4]. In 2021 rotator cuff tears were not included. The updated SAPS guideline focuses on degenerative rotator cuff pathology, a field with ongoing research into conservative and surgical treatment. This has yielded new insights into treatment modalities, as well as postoperative rehabilitation.

**Table:** This table summarizes the clinical questions addressed in Part 2 of the guideline, along with the outcomes measured for each module

Clinical topic	Scoping questions	Outcomes
1. Cuff repair vs physiotherapy		
What is the effectivity of cuff repair compared with physiotherapy with or without corticosteroid injection on patient-reported outcome measures in adult patients (< 70 years) with an isolated symptomatic, nontraumatic, supraspinatus tear?		Pain, PROMs for function (CMS, DASH, WORC, ASES, DSST, OSS), complications (re-tear), patient satisfaction, return to work or leisure
2. Long head biceps tendon treatment		
What are the effects of tenotomy or tenodesis of the LHBT in patients with an isolated supraspinatus tendon tear and no LHBT pathology?		Pain, PROMs for function (CMS, DASH, WORC, ASES, OSS), complications (Popeye sign), patient satisfaction, return to work or leisure
3. Prognostic factors		
What are the prognostic factors for success or failure after rotator cuff repair?		Predictive value/model performance: 0.7≤AUC<0.8 (acceptable), 0.8≤AUC<0.9 (excellent), AUC≥0.9 (outstanding)
4. Postoperative immobilization		
What is the effectiveness of early mobilization supported by a sling up to max. 3 weeks, vs long-term (6 weeks) immobilization with a sling in patients where the supraspinatus tendon is repaired?		Pain, PROMs for function (CMS, DASH, WORC, ASES, DSST, OSS, postoperative stiffness), frozen shoulder, complications (re-tear), patient satisfaction, return to work or leisure
5. Surgical calcium removal vs barbotage		
What is the effectiveness of surgical calcium removal compared with barbotage on patient-reported outcome measures in adult patients with calcifying tendinitis of the supra- or infraspinatus?		Pain, PROMs for function (CMS, DASH, WORC, ASES, OSS, DSST), complications, patient satisfaction, return to work or leisure

LHBT: long head biceps tendon; PROMs: patient-reported outcome measure; CMS: Constant-Murley Score; DASH: Disabilities of the Arm, Shoulder and Hand; WORC: Western Ontario Rotator Cuff Index; ASES: American Shoulder and Elbow Surgeons Score; DSST: Dutch Simple Shoulder Test; OSS: Oxford Shoulder Score; AUC: area under the curve; MRI: magnetic resonance imaging.

For this part 2 the guideline development group formulated 4 key questions addressing rotator cuff pathology, including indications and prognostic factors for surgery for patients with degenerative supraspinatus tendon tears, the treatment of the long head biceps tendon (LHBT) in patients with a rotator cuff tear, postoperative rehabilitation following cuff repair, and a comparison of surgical treatment for calcifying tendinitis with needle aspiration of the calcific depot (NACD). This guideline is designed for orthopedic surgeons, sports medicine specialists, physiotherapists, and rehabilitation doctors involved in the care of patients with SAPS. It also aims to inform other healthcare providers engaged in treating these patients, including physicians, general practitioners, anesthesiologists, radiologists, rheumatologists, physician assistants, and nurse practitioners.

## Methods

See the Dutch Guideline on treatment of SAPS Part 1 for the guideline methodology [1].

### Terminology

Rotator cuff tears present in various forms, with a primary distinction between traumatic and non-traumatic causes. Degenerative tears also differ in location and extent. The subscapularis tendon plays a key role in stabilizing the LHBT, and its tears are typically trauma-related [5]. Traumatic tears, such as those following anterior shoulder dislocation with good tissue quality, may warrant surgical repair. In multi-tendon injuries, (partial) cuff repair or tendon transfer can help restore function. Given the variability in tear patterns and treatment approaches, this guideline focuses specifically on isolated, symptomatic, non-traumatic supraspinatus tears

The table presents the clinical topic with corresponding clinical questions and the outcome measures used.

### Funding, use of AI, and disclosures

See part 1 [1].

## Results

### Clinical question 1: What is the effectivity of cuff repair compared with physiotherapy with or without corticosteroid injection on patient-reported outcome measures in adult patients (< 70 years) with an isolated symptomatic, nontraumatic supraspinatus tear?

A systematic literature search identified 8 publications [6-13] covering 4 primary studies. Due to risk of bias, the applicability of the results due to inclusion of traumatic tears, and small sample sizes, the quality of the studies was rated low to very low. The evidence on the effectiveness of rotator cuff repair vs physiotherapy (including or excluding corticosteroid injections) on function (measured by Constant-Murley score [14] and American Shoulder and Elbow Surgeons Score [15]) at 1, 2, 5, or 10 years is very uncertain. Non-surgical treatment, involving 6–9 months of physical therapy, is less costly compared with surgery. Success rates for conservative treatment vary, with reported effectiveness around 75% [13,16]. Surgery remains an option if symptoms persist after 3–6 months of non-surgical treatment [17].

***Recommendations:***

- For patients with an isolated, symptomatic, non-traumatic supraspinatus tendon tear, start with non-surgical therapy, such as exercise therapy, potentially combined with a corticosteroid injection.
- Consider surgical treatment if non-surgical therapy is not effective after 3–6 months.

### Clinical question 2: What are the effects of tenotomy or tenodesis of the LHBT in patients with an isolated supraspinatus tendon tear and no LHBT pathology?

The systematic search yielded 276 hits, but no studies met the inclusion criteria. No conclusions could be drawn from the literature. Recommendations are based on expert opinion. For patients with pathology of the LHBT, both tenotomy and tenodesis are effective treatments with no clinically relevant differences.

***Recommendations:***

- Exercise caution when performing biceps tenotomy or tenodesis on a normal-looking LHBT during a supraspinatus tendon repair, because there is a risk of damaging the LHBT as a result of the repair.

### Clinical question 3: What are the prognostic factors for success or failure after rotator cuff repair?

The systematic search resulted in 829 hits, but no studies were included in the analysis, as no models predicting success or failure of surgical treatment for rotator cuff tears were reported. However, several factors influencing cuff integrity after repair have been identified in the literature [10,18-23]. These factors can be categorized into cuff-related factors, patient-related factors, and surgical technique (not covered in this guideline). Rehabilitation considerations are addressed in clinical question 4.

Cuff-related factors identified are tear size, fatty infiltration, muscle atrophy, suprascapular nerve neuropathy, tendon delamination, retraction, tear location, and shoulder stiffness. These factors are negatively correlated with outcome.

Patient-related factors identified are: age, smoking, BMI, diabetes mellitus, dyslipidemia, vitamin D deficiency, osteoporosis, and NSAID use. Corticosteroid injection 3–6 months before surgery may increase the risk of re-tear. These factors are negatively correlated with outcome.

***Recommendation:***

- Assess both patient- and cuff tear-related factors to estimate the success rate of rotator cuff repair.
- Consider performing MRI investigation of the shoulder for a more detailed evaluation of cuff tear-related factors.

### Clinical question 4: What is the effectiveness of early mobilization supported by a sling up to a maximum of 3 weeks vs long-term (6 weeks) immobilization with a sling in patients where the supraspinatus tendon is repaired?

The literature search resulted in 180 hits, with 3 studies [24-26] meeting the inclusion criteria. Evidence suggests that early mobilization supported by a sling for up to 3 weeks likely results in no significant difference in function at 6 months compared with long-term immobilization (moderate quality of evidence). No difference between the 2 groups was found in pain at 12 months. Early mobilization may reduce shoulder stiffness at 12 months’ follow-up (low-quality evidence). The studies were underpowered to assess a difference in re-tear rate. In another randomized controlled trial (RCT) with small to medium-sized rotator cuff tears no difference in re-tear rate was found between 3 and 6 weeks of immobilization [25]. Patients benefit from shorter immobilization periods.

***Recommendation:***

- Consider starting early mobilization with a maximum of 3 weeks’ immobilization in patients following surgical repair of an isolated supraspinatus tendon tear.

### Clinical question 5: What is the effectiveness of surgical calcium removal compared with barbotage on patient-reported outcome measures in adult patients with calcifying tendinitis of the supra- or infraspinatus?

The literature search resulted in 103 hits. 14 studies were selected based on title and abstract screening; eventually 1 study [27] met the inclusion criteria. Low-quality evidence was found that arthroscopic procedures result in little to no difference in pain (VAS) at 12 and 24 months. Surgery resulted in little to no difference in complications and adverse events compared with needle fragmentation of shoulder calcifications. The evidential strength for these crucial and important outcome measures is low. This is because only 1 study was included, with a low number of patients per study arm. An advantage of needle aspiration is that it can be performed in an outpatient setting under local anesthesia. The costs are lower compared with a surgical procedure.

***Recommendation:***

- Start with conservative treatment for symptomatic calcifying tendinitis, given its self-limiting nature.
- Consider barbotage if conservative treatment fails, acknowledging limited evidence for additional benefit over corticosteroid injection alone.
- Reserve arthroscopic treatment for cases with persistent, disabling symptoms and large calcifications after failed conservative treatment, including barbotage.

## Discussion

The goal of this guideline is to decrease variation in clinical practice, improve clinical outcomes, and support shared decision-making. It was developed to inform all healthcare professionals involved in managing SAPS, including orthopedic surgeons, physical therapists, anesthesiologists, radiologists, and general practitioners.

### Surgical vs non-surgical treatment of rotator cuff tears

Rotator cuff tears present with a wide range of radiological and clinical variation, making direct comparisons in RCTs challenging. This complexity was reflected in the studies included, which were rated as having very low to low quality of evidence [6-13]. Based on the literature analysis, no definitive conclusions could be drawn regarding the effect of rotator cuff repair surgery on outcomes such as function, pain, quality of life, return to work and leisure activities, and complications, compared with exercise/physiotherapy. Non-surgical treatment is effective in approximately 75% of patients with isolated supraspinatus tendon tears [13,16]. On the other hand, there is evidence suggesting that delaying surgery for over a year after non-surgical treatment leads to worse outcomes compared with a delay of 3–6 months [17]. Given the high success rate of non-surgical treatment, the lack of short- and medium-term differences between conservative and surgical approaches, the lower cost of non-surgical treatment, and the option of surgery after 3 months of nonoperative management, the working group recommends non-surgical therapy for patients below the age of 70 with an isolated, symptomatic, non-traumatic supraspinatus tear. Patients should be informed of the potential risk of tear progression with non-surgical treatment, and follow-up should be conducted [13]. If no improvement occurs after 3–6 months, rotator cuff repair may be considered.

### Prognostic factors for surgical decision-making

No predictive models for surgical success or failure in rotator cuff tears were found in the literature, highlighting a knowledge gap. Although no validated prediction models exist for determining the success of rotator cuff repair, many factors are known that can guide the advice given to patients with a rotator cuff tear. The literature describes several factors that are important for cuff integrity and clinical outcomes following surgical treatment. The scientific evidence comes from a wide range of studies: prospective studies, retrospective studies, multivariate analyses, animal studies, underpowered studies, studies with different outcome measures, and so on [18]. These factors can be categorized into 3 groups: (i) cuff tear-related factors; (ii) patient-related factors; (iii) surgical repair technique and postoperative rehabilitation. The surgical repair technique is beyond the scope of this guideline. Clinicians should assess patient- and tear-related prognostic factors to evaluate the likelihood of surgical success. The advantages and disadvantages of rotator cuff repair should be discussed with patients to facilitate shared decision-making.

### Long head biceps tendon management in rotator cuff repair

A literature review assessed the effect of tenotomy or tenodesis of the LHBT vs no intervention in patients with a normal-appearing LHBT undergoing supraspinatus repair. No systematic reviews, RCTs, or comparative observational studies met the PICO criteria, so no studies were included in the literature summary. A systematic review and meta-analysis shows that both LHBT tenotomy and tenodesis lead to good results, with no clinically relevant differences [28]. Treating the LHBT carries procedural risks for damage during supraspinatus repair. Based on the existing literature, it is unclear whether there is any added benefit to performing an additional LHBT tenotomy or tenodesis on an LHBT that appears normal. Potential LHBT treatment options that may be performed intraoperatively should be discussed with the patient beforehand. Biceps pathology cannot always be ruled out beforehand, and tenodesis or tenotomy may therefore become necessary.

### Postoperative rehabilitation

The goal of rotator cuff repair is to restore shoulder function, alleviate pain, and improve strength by reattaching the torn tendon to the greater tubercle, promoting healing, and preventing further degeneration or re-tears. This outcome was assessed in only 1 underpowered study that evaluated re-tear rates for different periods of immobilization [25]. In this RCT, no difference was found in re-tear rates for small- to medium-sized rotator cuff tears between 3- and 6-week immobilization periods. However, a systematic review subgroup analysis reported significantly more re-tears after early mobilization in large rotator cuff tears [29]. Patients benefit from early mobilization [30,31]; therefore, we advise considering immobilizing the shoulder for a maximum period of 3 weeks after surgical repair of an isolated small- to medium-sized supraspinatus tendon rupture.

### Calcific deposit removal: surgical vs conservative approach

Only 1 study was included comparing surgical removal of a calcium deposit vs needle aspiration [27]. Based on low-quality evidence, arthroscopic removal resulted in little to no difference in pain or complications compared with needle fragmentation at 12 and 24 months. A knowledge gap remains: what is the effectiveness of surgical calcium removal compared with barbotage on patient-reported outcome measures in adult patients with supra- or infraspinatus calcifying tendinitis?

Given that calcific tendinitis is a self-limiting disease, conservative treatment is recommended as the initial approach. The effectiveness of barbotage remains under debate. A recent sham-controlled randomized controlled trial found no long-term difference between barbotage and sham treatment, suggesting limited added value [32]. However, these results conflict with findings from another RCT, which reported significant benefits of barbotage after 1 year [33]. The discrepancy may be explained by differences in follow-up duration, the technical success of complete calcification removal, and the natural history of calcific tendinitis as a self-limiting condition [32,34].

As barbotage is less invasive than surgical removal and can be performed in an outpatient setting under local anesthesia with lower costs, barbotage is preferred over surgical treatment. However, given the limited evidence for additional benefit over corticosteroid injection alone and the self-limiting nature of the disease, patients should be informed that conservative treatment with observation may be equally effective in many cases. The effectiveness of surgical calcium removal after failed barbotage remains unclear.

### Perspectives

This guideline was composed for managing SAPS. In the management of SAPS there is still debate and controversy on the optimal treatment per patient. This guideline aims to support healthcare providers and patients in shared decision-making for SAPS management. Part 2 specifically addresses the surgical aspects of SAPS, including patient selection and rehabilitation.

### Supplementary data

Work group details, conflict of interests, search strategies and evidence tables are available as supplementary data on the article page of Part 1 [1].

## Notes

Handling co-editor: Jeppe Vejlgaard Rasmussen

*Acta* thanks Teemu Karjalainen and Adam Witten for help with peer review of this manuscript.
